# Social Precariousness and the Outcome of Critical Illnesses in People with HIV: A Multicenter Cohort Study

**DOI:** 10.1093/ofid/ofaf687

**Published:** 2025-11-12

**Authors:** Piotr Szychowiak, Thierry Boulain, Étienne de Montmollin, Jean-François Timsit, Alexandre Elabbadi, Laurent Argaud, Stephan Ehrmann, Nahema Issa, Emmanuel Canet, Frédéric Martino, Fabrice Bruneel, Jean-Pierre Quenot, Florent Wallet, Élie Azoulay, François Barbier

**Affiliations:** Médecine Intensive Réanimation, Centre Hospitalier Universitaire d’Orléans, Orléans, France; Médecine Intensive Réanimation, Centre Hospitalier Universitaire d’Orléans, Orléans, France; Réanimation Médicale et des Maladies Infectieuses, Centre Hospitalier Universitaire Bichat-Claude Bernard, Assistance Publique-Hôpitaux de Paris, Paris, France; Réanimation Médicale et des Maladies Infectieuses, Centre Hospitalier Universitaire Bichat-Claude Bernard, Assistance Publique-Hôpitaux de Paris, Paris, France; Médecine Intensive Réanimation, Centre Hospitalier Universitaire Tenon, Assistance Publique-Hôpitaux de Paris, Paris, France; Médecine Intensive Réanimation, Centre Hospitalier Universitaire Edouard Herriot, Hospices Civiles de Lyon, Lyon, France; Médecine Intensive Réanimation, INSERM CIC 1415 et CRICS-TriggerSEP F-CRIN Research Network, Centre Hospitalier Universitaire de Tours and Centre d’Etude des Pathologies Respiratoires, INSERM U1100, Université de Tours, Tours, France; Médecine Intensive Réanimation, Centre Hospitalier Universitaire de Bordeaux, Bordeaux, France; Médecine Intensive Réanimation, Centre Hospitalier Universitaire de Nantes, Nantes, France; Médecine Intensive Réanimation, Centre Hospitalier Universitaire de la Guadeloupe, Pointe-à-Pitre, France; Médecine Intensive Réanimation, Centre Hospitalier de Versailles, Le Chesnay, France; Médecine Intensive Réanimation, Centre Hospitalier Universitaire de Dijon-Bourgogne, Dijon, France; Médecine Intensive Réanimation, Centre Hospitalier Universitaire Lyon Sud, Hospices Civiles de Lyon, Lyon, France; Médecine Intensive Réanimation, Centre Hospitalier Universitaire Saint-Louis, Assistance Publique-Hôpitaux de Paris, Paris, France; Médecine Intensive Réanimation, Centre Hospitalier Universitaire d’Orléans, Orléans, France

## Abstract

**Background:**

Social precariousness hinders access to the cascade of care in people with HIV (PHIV). Its impact on the clinical presentation and outcome of critical illnesses in this patient population is unknown.

**Methods:**

We included all PHIV admitted over the 2015 to 2020 period in 12 university-affiliated intensive care units in France. Precarious patients encompassed undocumented migrants, homeless, and individuals facing other forms of socioeconomic deprivation. Precarious and nonprecarious PHIV were compared for baseline characteristics and reasons for admission. The effect of precariousness on in-hospital mortality (primary endpoint) and 1-year mortality (secondary endpoint) was measured through logistic regression.

**Results:**

Among the 939 included PHIV, 136 (14.5%) were classified as precarious (migrants, 5.7%; others, 8.7%). Compared to nonprecarious patients, (1) migrants were younger, had fewer comorbidities, and were more often admitted with previously unknown HIV and/or for AIDS-defining opportunistic infections; and (2) precarious patients other than migrants presented with lower rates of viral suppression (despite similar access to combination antiretroviral therapies) and were more often admitted for bacterial sepsis. Overall in-hospital and 1-year mortality rates were 17.8% and 24.2%, respectively. Precariousness was not independently associated with in-hospital mortality (adjusted odds ratio, 1.04; 95% confidence interval, .98-1.10) or 1-year mortality (adjusted odds ratio, .89; 95% confidence interval, .54-1.48), including when analyzing migrants separately.

**Conclusions:**

Precarious PHIV requiring intensive care unit admission have particular clinical features that likely reflect chronic inequities in access to HIV care. However, precariousness is probably not linked with a higher hazard of death during the index hospital stay or at 1 year.

The clinical features and outcomes of people living with HIV (PHIV) admitted to the intensive care unit (ICU) evolve owing to improved access to the continuum of HIV care, from early diagnosis to sustained viral suppression and immune recovery under combination antiretroviral therapy (cART) [1]. Main trends include aging resulting from extended life expectancy, a rising prevalence of cancer and other comorbidities that may be either associated or not with chronic HIV, a marked drop in inaugural and/or AIDS-related admissions, and a substantial increase in short-term survival rates that also ensues from general advances in ICU practices [2–5].

Yet, the availability of dedicated health services remains heterogeneous among PHIV and individuals facing extreme poverty, isolation, or other forms of social insecurity still constitute a particularly vulnerable subgroup [6, 7]. Evidence has accumulated over the recent years that social precariousness correlates with stigma and discriminations in access to care, delayed testing, and impaired retention in all components of long-term HIV management, including cART adherence [8–13]. These impediments to the successful control of HIV infection appears exacerbated in undocumented migrants [14, 15], a population at high risk for both pre- and postmigration HIV acquisition and which represents an emerging profile of PHIV in Western countries due to the ongoing humanitarian crises and refugee displacements [16–18].

The prognostic effect of precariousness remains equivocal in the general population of ICU patients and has not been specifically investigated in those with HIV infection [19–21]. Whether chronic restrictions in access to specialized care may impact the clinical presentation and outcomes of critical illnesses in precarious PHIV is unknown. The objectives of this multicenter retrospective cohort study were to assess whether social precariousness is associated with in-hospital mortality (primary endpoint) and 1-year mortality (secondary endpoint) in PHIV requiring ICU admission. Baseline characteristics and reasons for admission were also investigated. Subgroup analyses were conducted in undocumented migrants and other precarious patients.

## PATIENTS AND METHODS

### Study Design

This study was conducted in 12 medical ICUs located in university-affiliated hospitals in France and contributing to the Groupe de Recherche Respiratoire en Réanimation Onco-Hématologique (www.grrroh.fr) and Clinical Research in Intensive Care and Sepsis—Trial Group for Global Evaluation and Research in Sepsis (www.crics-triggersep.org) networks for research in intensive care. All adult PHIV admitted to these ICUs over a 5.5-year period (from January 2015 to June 2020) were retrospectively identified by local investigators through hospital coding databases using the International Classification of Diseases—10th revision items related to HIV (that is, B20-24, Z21, and R75) and considered for inclusion in the study (see the following section). This cohort was previously exploited to investigate the clinical presentation and outcomes of cancer in critically ill PHIV [3]. The study protocol was approved by the ethical committee of the French Intensive Care Society on 3 August 2020 (report no. CE-SRLF-20-70); the requirement for informed consent was waived owing to the observational design. Results are reported according to the STROBE guidelines (www.strobe.org).

### Patient Selection, Data Collection, and Definitions

The patient medical records from the ICU and the downstream unit were anonymized by local investigators and centralized before data extraction by 2 of the study coordinators (P.S. and F.B.) using a standardized form. Patients with missing information regarding cART use at ICU admission and the vital status at hospital discharge were excluded from the study population after medical chart reviewing. Only the first ICU stay was analyzed in patients with multiple admissions over the inclusion period.

Patients were classified as precarious if they were (1) undocumented migrants or (2) homeless, incarcerated, or experiencing other forms of socioeconomic deprivation such as social marginalization, precarious housing, and extreme poverty [22]. Details regarding the classification process are provided in the electronic supplement.

Data related to demographics, characteristics of HIV, chronic conditions other than AIDS, reasons for ICU admission, use of organ support, treatment limitation decisions, lengths of stay, and hospital and 1-year mortality were extracted from the medical charts. cART was defined according to the current International Antiviral Society guidelines [23]. When available, CD4 cell count and HIV viral load were collected within the 6 months preceding ICU admission for patients with previously known HIV and at ICU admission for those with newly diagnosed seropositivity. AIDS-defining conditions were defined according to the Centers for Diseases Control and Prevention classification. AIDS-defining and non-AIDS-defining cancers (ADC/NADC) were considered when active (ie, inaugural admission, response to first-line therapy, relapse, or refractory disease) or in remission for less than 5 years.

The primary diagnoses of the ICU stay were ventilated as bacterial sepsis, AIDS-defining opportunistic infections, AIDS-defining cancer, non-AIDS-defining cancer, exacerbation of non-AIDS-related comorbidities (other than cancer), and others.

To investigate long-term survival after hospital discharge, local investigators collected and provided the vital status of included patients at last visit in each participating center. Patients without consultation or readmission in the participating centers were considered as lost to follow-up. Information regarding subsequent deaths not occurring in participating hospitals was not available.

The primary study endpoint was in-hospital mortality. The secondary study endpoint was mortality at 1 year following ICU admission.

### Statistical Analyses

Data are expressed as median (interquartile range) for continuous variables and number (percentage) for categorical variables, unless otherwise indicated. The analysis of variance test and the Kruskal-Wallis test were used to compare continuous variables with normal and non-normal distributions, respectively. Categorical variables were compared using the Fisher exact test or the χ^2^ test, as appropriate.

Odds ratios (OR) and their 95% confidence interval (CI) for the reasons of ICU admission in precarious versus nonprecarious patients were calculated after adjustment on age and sex.

The cumulative survival at 1 year following ICU admission was compared between precarious and nonprecarious patients through Kaplan-Meier analyses and the log-rank test, with right-censoring at the date of last follow-up information.

To assess the patient characteristics that could have influenced the occurrence of the study endpoints, we used a logistic regression model with in-hospital death or death at 1 year as the dependent variable. As independent variables, we first entered in a global starting model a number of patient characteristics that could have been linked to these endpoints (see the electronic supplement). To reduce the number of potential independent variables per event, we then proceeded to augmented backward elimination that combines the standardized change-in-estimate criterion with significance-based backward elimination and liberal criteria to keep independent variables in the model (threshold values, *P* < .35, and change-in-estimate <35%) to minimize the risk of eliminating important explanatory variables [24]. Passive variables that were considered systematically associated with the study endpoints (namely age and the sepsis-related organ failure assessment score value at ICU admission) were kept in the models. Other active variables that could have been linked to in-hospital or 1-year mortality or have modified the influence of passive variables were also introduced in the starting model submitted to augmented backward elimination. Precariousness was forced into the models. This procedure was repeated on 2000 nonstratified bootstrap replicates of the study population. Potentially explanatory variables (exposed with their adjusted OR [aOR] and 95% CI) were retained in the final models if they were selected in more than 50% of the bootstrap samples, with a root mean square difference ratio <1.5 and an absolute relative conditional bias <50%. Multicollinearity was searched for through calculation of the variance inflation factor (VIF) for each independent variable. Discriminative ability of the models was assessed through area under the receiver operating characteristics curve (AUROC). The same approach was used for subgroup analyses restricted to undocumented migrants.

Analyses were conducted using the R software version 4.2.3 (http://www.R-project.org). Two-tailed *P* values <.05 were considered statistically significant.

## RESULTS

### Study Population and Baseline Features of Patients With Social Precariousness

A total of 939 PHIV were included in the study cohort (Table 1, and Supplementary Figure 1 in the electronic supplement). Among them, 136 (14.5%) were classified as precarious, including 54 undocumented migrants (5.8%) and 82 patients other than undocumented migrants (8.7%) (homeless, n = 29 [3.1%]; incarcerated, n = 1 [0.1%]; other forms of social deprivation, n = 52 [5.5%]) (Supplementary Table 1). These proportions did not differ significantly across centers (*P* = .80, Supplementary Figure 2).

**Table 1.** Characteristics of the Study Population

Characteristics	All Patients (n = 939)	Nonprecarious Patients (n = 803)	Precarious Patients (n = 136)	*P* Value
Male sex	670 (71.3)	582 (72.5)	88 (64.7)	.08
Age, y	52 (43–59)	53 (44–60)	45 (36–55)	<.001
**Addiction (current or past)**				
Tobacco	417 (44.4)	366 (45.6)	51 (37.5)	.08
Alcohol	176 (18.7)	148 (18.4)	28 (20.6)	.55
Drug	180 (19.2)	148 (18.4)	32 (23.5)	.03
**Chronic conditions**				
Any	745 (79.3)	646 (80.4)	99 (72.8)	.04
Diabetes mellitus	134 (14.3)	124 (15.4)	10 (7.4)	.02
Respiratory	199 (21.2)	176 (21.9)	23 (16.9)	.23
COPD	107 (11.4)	93 (11.6)	14 (10.3)	.77
Cardiac	175 (18.6)	162 (20.2)	13 (9.6)	.005
Hepatic	200 (21.3)	173 (21.5)	27 (19.9)	.97
HBV co-infection	72 (7.7)	62 (7.7)	10 (7.4)	1
HCV co-infection	111 (11.8)	92 (11.5)	19 (14.0)	.49
Liver cirrhosis	74 (7.9)	66 (8.2)	8 (5.9)	.44
Renal	171 (18.2)	150 (18.7)	21 (15.4)	.95
Chronic hemodialysis	30 (3.2)	25 (3.1)	5 (3.7)	.79
Neurological	105 (11.2)	90 (11.2)	15 (11.0)	1
Psychiatric	114 (12.1)	89 (11.1)	25 (18.4)	.004
AIDS-defining cancer^a^	106 (11.3)	90 (11.2)	16 (11.8)	.88
Solid	20 (2.1)	16 (1.7)	4 (2.9)	.51
Hematological	86 (9.2)	74 (9.2)	12 (1.3)	1
Non-AIDS-defining cancer^a^	97 (10.3)	81 (10.1)	16 (11.8)	.54
Solid	62 (6.6)	52 (6.5)	10 (1.1)	.71
Hematological	35 (3.7)	29 (3.6)	6 (4.4)	.62
**WHO performance status**				
0–1	848 (90.3)	722 (89.9)	126 (92.7)	.32
2–4	91 (9.7)	81 (10.1)	10 (7.3)	
**HIV-related characteristics**				
Newly diagnosed HIV	127 (13.5)	103 (12.8)	24 (17.6)	.17
CD4+ cell count at admission, per µL^b^	51 (20–147)	50 (20–141)	70 (23–181)	.46
Previously known HIV infection	812 (86.5)	700 (87.2)	112 (82.4)	.17
Previous AIDS-defining disease	349 (37.2)	292 (36.4)	57 (41.9)	.25
cART at hospital admission	699 (74.4)	613 (76.3)	86 (63.2)	<.001
Baseline CD4+ cell count, per µL^c^	370 (180–600)	381 (199–600)	251 (105–552)	.01
Virally controlled	772 (82.2)	674 (83.9)	98 (72.1)	<.001
**SAPS-2 at ICU admission**	36 (26–51)	36 (26–50)	35 (26–51)	.65
**SOFA score at ICU admission**	4 (2–6)	4 (2–7)	3 (1–6)	.33
**Mode of ICU admission**				
Direct from the emergency department	603 (64.2)	518 (64.5)	85 (62.5)	.70
Transfer from hospital wards	336 (35.8)	285 (35.5)	51 (37.5)	
**Main reason for ICU admission**				
Acute respiratory failure	320 (34.1)	283 (35.2)	37 (27.2)	.07
Sepsis/septic shock	172 (18.3)	140 (17.4)	32 (23.5)	.05
Coma (nontoxic)	165 (17.6)	137 (17.1)	28 (20.6)	.22
Acute kidney injury	56 (6.0)	50 (6.2)	6 (4.4)	.41
Metabolic	47 (5.0)	39 (4.9)	8 (6.9)	.61
Drug overdose	47 (5.0)	37 (4.6)	10 (7.4)	.17
Shock (other than septic)	28 (3.0)	26 (3.2)	2 (1.5)	.41
Cardiac arrest	16 (1.7)	14 (1.7)	2 (1.5)	1
Others	88 (9.4)	77 (9.6)	11 (8.1)	.75
**Type of ICU admission**				
Medical	904 (96.3)	771 (96.0)	133 (97.8)	.59
Unscheduled surgery	13 (1.4)	12 (1.5)	1 (0.7)	
Scheduled surgery	22 (2.3)	20 (2.5)	2 (1.5)	
**Organ support during the ICU stay**				
Invasive mechanical ventilation	301 (32.1)	260 (32.4)	41 (30.1)	.69
Vasopressors	242 (25.8)	212 (26.4)	30 (22.1)	.34
Renal replacement therapy	105 (11.2)	93 (11.6)	12 (8.8)	.38
ECMO	14 (1.5)	14 (1.7)	0	.24
**Main diagnosis of the ICU stay**				
Bacterial sepsis	263 (28.0)	219 (27.3)	44 (32.4)	.22
AIDS-defining opportunistic infection	156 (16.6)	125 (15.6)	31 (22.8)	.05
Tuberculosis^d^	14 (1.5)	6 (0.7)	8 (6.9)	<.001
Cerebral toxoplasmosis	30 (3.2)	20 (2.5)	10 (7.4)	.001
*Pneumocystis jirovecii* pneumonia	69 (7.3)	63 (7.8)	6 (4.4)	.16
Cryptococcosis^d^	10 (1.1)	7 (0.9)	3 (2.2)	.17
Others	33 (3.5)	29 (3.6)	4 (2.9)	1
AIDS-defining cancer	69 (7.3)	62 (7.7)	7 (5.1)	.37
Non-AIDS-defining cancer	47 (5.0)	42 (5.2)	5 (3.7)	.53
Exacerbation of chronic conditions other than cancer	242 (25.8)	207 (25.8)	34 (25.0)	.91
Miscellaneous	162 (17.3)	147 (18.3)	15 (11.0)	.04
**MDRB carriage at ICU admission**	121 (12.9)	96 (12.0)	25 (18.4)	.04
ESBL-producing Enterobacterales	102 (10.9)	82 (10.2)	20 (14.7)	.12
**Treatment limitation decisions**	110 (11.7)	91 (11.3)	19 (14.0)	.38
**Outcomes**				
Length of stay in the ICU, days	5 (3–9)	5 (3–9)	4 (3–8)	.26
Length of stay in the hospital, d	19 (10–36)	19 (10–35)	18 (8–45)	.25
In-ICU mortality	112 (11.9)	96 (12.0)	16 (11.8)	.95
In-hospital mortality	167 (17.8)	142 (17.7)	25 (18.4)	.84
1-y mortality^e^	227/744 (30.5)	196/637 (30.8)	31/107 (29.0)	.73
1-y mortality in patients discharged alive from the hospital	60/577 (10.4)	54/495 (10.9)	6/82 (7.3)	.43

Data are exposed as number (%) or median (interquartile range).

Abbreviations: cART, combination antiretroviral therapy; COPD, chronic obstructive pulmonary disease; ECMO, extracorporeal membrane oxygenation; ESBL, extended-spectrum β-lactamase; HBV/HCV, hepatitis B/C virus; ICU, intensive care unit; MDRB, multidrug-resistant bacteria; SAPS, simplified acute physiology score; SOFA, sepsis-related organ failure assessment; WHO, World Health Organization.

^a^Active or remission <5 y.

^b^Missing values, n = 16.

^c^Missing values, n = 273.

^d^All presentations pooled.

^e^Vital status at 1 y was available for 577/772 survivors of the index hospital admission, including 82/111 precarious patients and 495/661 nonprecarious patients.

Undocumented migrants were younger, were mostly female, had less non-AIDS-related chronic conditions, and were far more often admitted with previously unknown HIV infection (38.9% vs 12.8% in nonprecarious patients, respectively; *P* < .0001) (Table 2). In this subgroup, only 50.0% of patients were receiving cART at the time of hospital admission, compared to 76.3% of nonprecarious patients (*P* < .0001).

**Table 2.** Key Features and Outcomes of Undocumented Migrants and Other Precarious Patients

Characteristics	Nonprecarious Patients (n = 803)	Precarious Patients (n = 136)			
		Migrants (n = 54)	*P* value^a^	Others (n = 82)	*P* value^a^
Male sex	582 (72.5)	25 (46.3)	.0001	63 (76.8)	.44
Age, y	53 (44–60)	40 (32–53)	<.0001	49 (40–56)	.008
Chronic conditions, any	646 (80.4)	32 (57.4)	.001	67 (81.7)	.88
**HIV-related characteristics**					
Newly diagnosed HIV	103 (12.8)	21 (38.9)	<.0001	3 (3.7)	.01
CD4+ cell count at admission, per µL	50 (20–141)	70 (22–193)	.55	79 (NA)	NA
Previously known HIV	700 (87.2)	33 (61.1)	<.0001	79 (94.3)	.01
History of AIDS-defining disease	292 (36.4)	15 (27.8)	.55	42 (51.2)	.01
cART at hospital admission	613 (76.3)	27 (50.0)	<.0001	59 (72.0)	.41
Baseline CD4+ cell count, per µL	381 (199–600)	293 (126–475)	.09	220 (84–600)	.03
Virally controlled	674 (83.9)	14 (25.9)	.001	34 (41.5)	<.0001
SOFA score at ICU admission	4 (2–7)	4 (2–6)	.85	3 (1–6)	.15
**Main diagnosis of the ICU stay**					
Bacterial sepsis	219 (27.3)	13 (24.1)	.06	31 (37.8)	.04
AIDS-defining opportunistic infection	125 (15.6)	17 (31.5)		14 (17.1)	
AIDS-defining cancer	62 (7.7)	3 (5.6)		4 (4.9)	
Non-AIDS-defining cancer	42 (5.2)	4 (7.4)		1 (1.2)	
Exacerbation of chronic condition other than cancer	207 (25.8)	9 (16.7)		25 (30.5)	
Miscellaneous	147 (18.3)	8 (14.8)		7 (8.5)	
Treatment limitation decision	91 (11.3)	6 (11.1)	1	13 (15.9)	.21
**Outcomes**					
ICU length of stay, d	5 (3–9)	5 (3–11)	.25	4 (2–6)	.02
Hospital length of stay, d	19 (10–35)	26 (15–52)	.01	14 (5–37)	.06
In-ICU mortality	96 (12.0)	9 (16.7)	.29	7 (8.5)	.47
In-hospital mortality	142 (17.7)	13 (24.1)	.27	12 (14.6)	.54
1-y mortality^b^	196/637 (30.8)	13/42 (30.9)	1	18/65 (27.7)	.67

Data are exposed as number (%) or median (interquartile range).

Abbreviations: CART, combination antiretroviral therapy; ICU, intensive care unit; SOFA, sepsis-related organ failure assessment.

^a^Comparisons with nonprecarious patients;.

^b^Vital status at 1 year was available for 577/772 survivors of the index hospital admission, including 29/41 undocumented migrants, 53/70 precarious patients other than migrants, and 495/66 nonprecarious patients.

Demographics, the prevalence of chronic conditions and prior cART exposure were similar in precarious patients other than migrants and nonprecarious patients (Table 2); however, a history of AIDS-defining diseases was more commonly observed in precarious patients other than migrants (51.2% vs 36.4%, *P* = .01).

Among patients known as HIV-infected before hospital admission, the baseline CD4 cell count was lower in precarious individuals than in their nonprecarious counterparts (251 [105–552] vs 381 [199–60] per mm^3^, *P* = .01), without difference between undocumented migrants and other precarious patients (293 [126–475] vs 220 [84–600] per mm^3^, *P* = .94). Viral control was less frequently observed in precarious than nonprecarious patients (Tables 1 and 2).

### Characteristics of the ICU Stay

Most patients were admitted directly from the emergency department. Acute respiratory failure, sepsis, and coma were the most common reasons for ICU admissions, without significant difference between precarious and nonprecarious patients (Table 1). Sepsis-related organ failure assessment score values at admission and the use of organ support during the ICU stay were similar in both groups.

**Figure 1.**
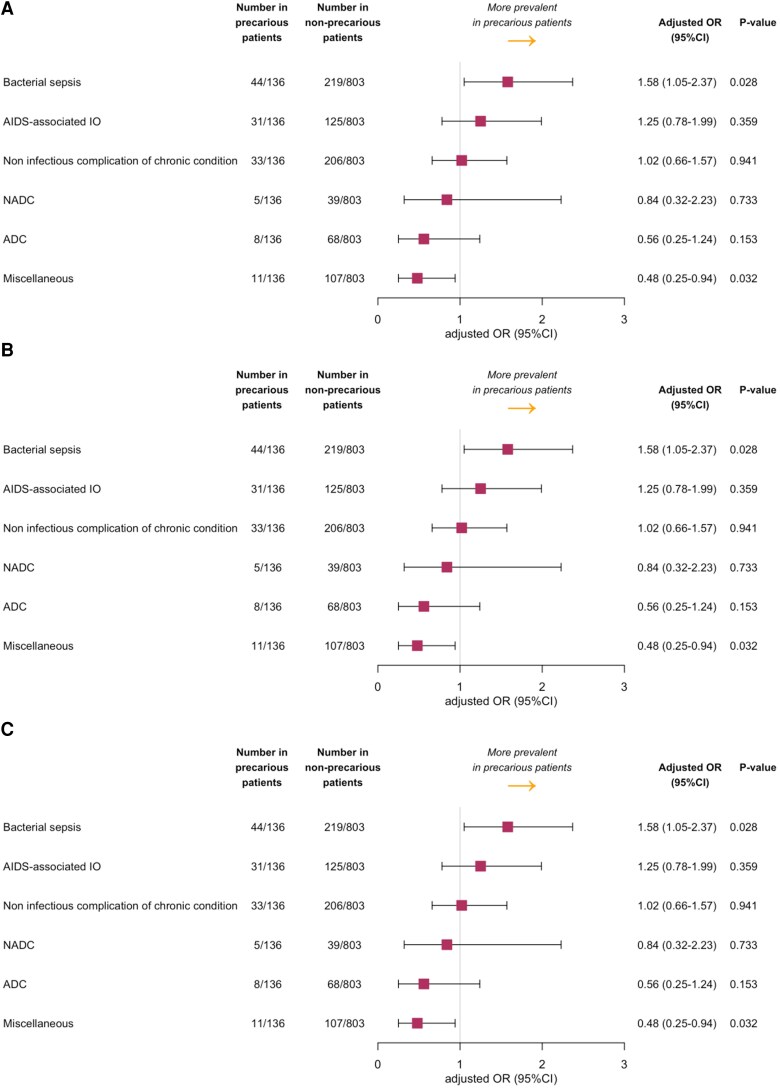
Odds ratio of the main diagnoses of the ICU stay in precarious versus nonprecarious critically ill patients with HIV after adjustment on age and sex. CI, confidence interval; ICU, intensive care unit; OR, odd ratio. (*A*) Precarious patients (all) versus nonprecarious patients. (*B*) Precarious patients other than migrants versus nonprecarious patients. (*C*) Undocumented migrants versus nonprecarious patients. ADC/NADC, AIDS-defining/non-AIDS-defining cancer; OI, opportunistic infection.

Main diagnoses of the ICU stay are exposed in Table 1. After adjustment on age and sex, admissions for bacterial sepsis were more common in precarious patients other than migrants (aOR, 1.89; 95% CI, 1.16–3.07; *P* = .01), whereas AIDS-defining opportunistic infections trended to be more prevalent in undocumented migrants (aOR, 1.86; 95% CI, .98–3.53; *P* = .06), cerebral toxoplasmosis being the most frequent presentation in this latter subgroup (Figure 1). Admissions for severe tuberculosis (all forms pooled) were more frequent in precarious patients than in their nonprecarious counterparts (6.9% vs 0.7%, *P* < .001), without significant difference between undocumented migrants and other precarious patients (Table 2). Admissions related to ADC, NADC, or exacerbations of chronic conditions other than cancer were equivalently observed in precarious and nonprecarious patients (Figure 1 and Table 1).

### Primary Study Endpoint: In-hospital Mortality

A total of 167 patients (17.8%) died during the index hospital admission. In-hospital mortality rates did not differ between precarious and nonprecarious patients (18.4% vs 17.7%, respectively; *P* = .84) (Table 1), as between undocumented migrants and other precarious patients (24.1% vs 14.6%; *P* = .18) (Table 2). No difference was observed between groups when appraising in-hospital mortality rates according to the main diagnoses of the ICU stay (Supplementary Figure 3). The full characteristics of survivors and deceased patients are compared in Supplementary Table 2. In multivariate regression analyses, precariousness was not independently associated with the likelihood of in-hospital death (aOR, 1.04; 95% CI, .98–1.10) (AUROC for model discriminability, 0.88; 95% CI, .85–.90) (Table 3). This lack of association was equally observed when analyzing migrants separately (aOR, 1.08; 95% CI, .99–1.19) (AUROC for model discriminability, 0.88; 95% CI, .85–.91) (**Table 3**). VIF values were <2.5 for all variables kept in these models, excluding significant multicollinearity.

**Table 3.** Independent Predictors of In-hospital Mortality: Results of Final Models

	All Patients		Migrants and Nonprecarious Patients Only	
	aOR (95% CI)	*P* value	aOR (95% CI)	*P* value
Male sex	.98 (.94–1.03)	.47	.98 (.93–1.03)	.41
Age, per 10-year increase	1.02 (.99–1.04)	.08	1.01 (.99–1.01)	.20
Alcohol addiction	.92 (.87-.97)	.002	.91 (.86-.96)	.001
Liver cirrhosis	1.12 (1.03–1.21)	.007	1.14 (1.05–1.24)	.002
Solid NADC	1.47 (1.35–1.61)	<.0001	1.44 (1.32–1.58)	<.0001
Hematological NADC	1.14 (1.02–1.27)	.025	1.11 (.99–1.25)	.08
WHO performance status	1.08 (1.02–1.14)	.012	1.08 (1.02–1.15)	.009
SOFA score value at ICU admission	1.03 (1.01–1.03)	<.0001	1.02 (1.01–1.03)	<.0001
Invasive MV during the ICU stay	1.05 (.99–1.12)	.09	1.05 (.98–1.12)	.15
ARDS during the ICU stay	1.26 (1.17–1.36)	<.0001	1.28 (1.18–1.38)	<.0001
Bacterial sepsis as main diagnosis of the ICU stay	.93 (.88-.98)	.009	.93 (.88-.98)	.01
Exacerbation of chronic condition as main diagnosis of the ICU stay	.93 (.88-.98)	.005	.93 (.88-.98)	.009
Newly diagnosed HIV	-	-	.97 (.91–1.04)	.43
History of AIDS-defining OI	-	-	.99 (.94–1.04)	.60
Vasopressor use during the ICU stay	-	-	1.06 (.99–1.15)	.09
RRT during the ICU stay	-	-	1.07 (.99–1.15)	.11
Precariousness	1.04 (.98–1.10)	.21	-	-
Precariousness (ie, undocumented migrants)	-	-	1.08 (.99–1.19)	.10

Abbreviations: aOR, adjusted odd ratio; CI, confidence interval; ADC/NADC, AIDS-defining/non-AIDS-defining cancer; ARDS, acute respiratory distress syndrome; ICU, intensive care unit; MV, mechanical ventilation; OI, opportunistic infection; RRT, renal replacement therapy; SOFA, sepsis-related organ failure assessment; WHO, World Health Organization.

### Secondary Study Endpoint: 1-year Mortality

**Figure 2.**
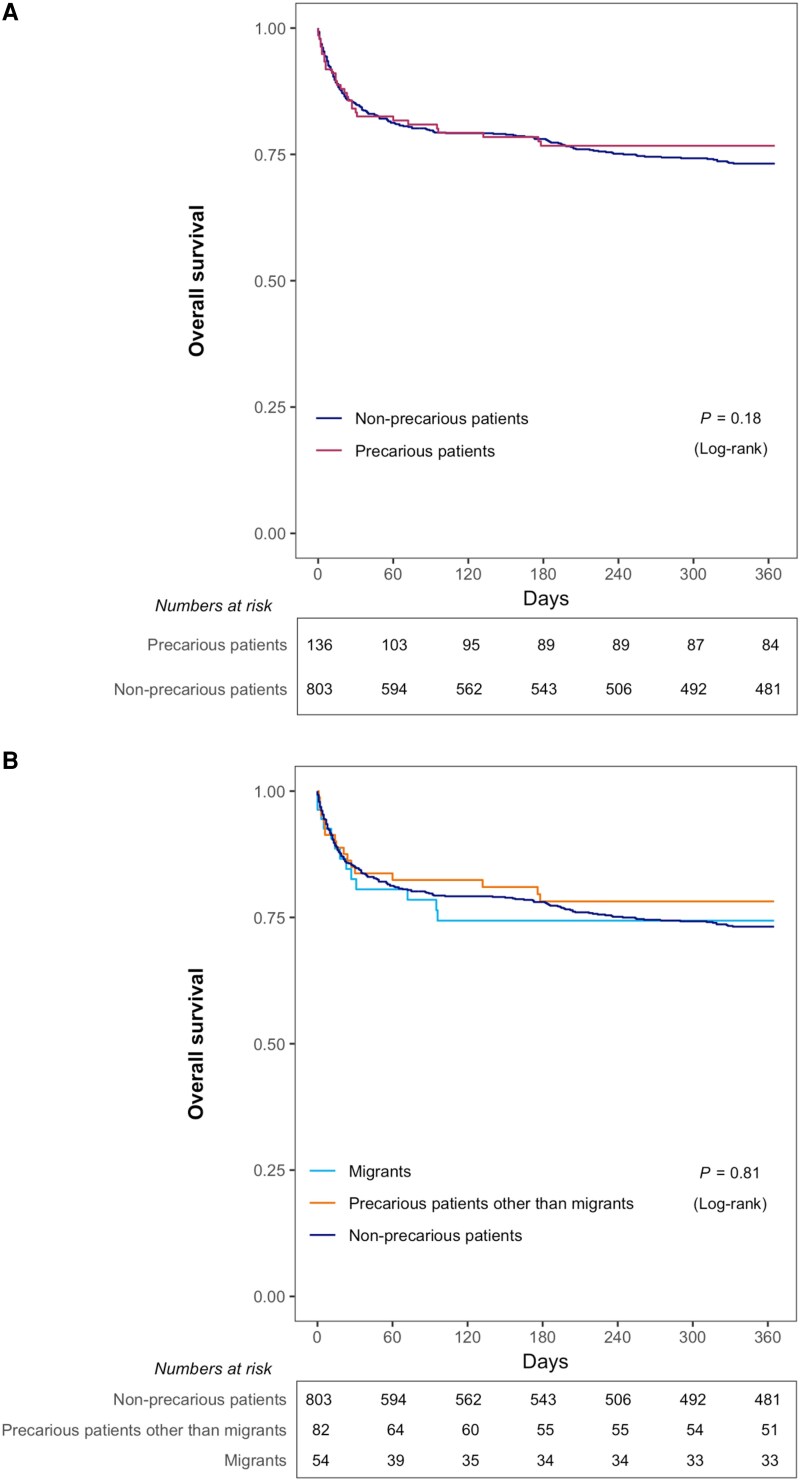
Cumulative 1-year survival in precarious and nonprecarious critically ill patients with HIV. D 0 indicates the date of admission to the intensive care unit. (*A*) Precarious patients (all) versus nonprecarious patients. (*B*) Precarious patients other than migrants versus undocumented migrants versus nonprecarious patients.

Vital status at 1 year was available for 577 (74.7%) of the 772 survivors of the index hospital admission, including 82/111 (73.9%) precarious patients and 495/661 (74.9%) nonprecarious patients. Cumulative 1-year mortality, assessed through Kaplan-Meier analyses with right-censoring at the time of last follow-up information, did not differ between precarious and nonprecarious patients (29.0% vs 30.8%, *P* = .18 by the log-rank test), including when analyzing undocumented migrants and precarious patients other than migrants separately (30.9% vs 27.7%, *P* = .81 by the log-rank test) (**Table 1**, **Table 2**, and **Figure 2**). In multivariate regression analyses, precariousness was not independently associated with 1-year mortality (aOR, 0.89; 95% CI .54–1.48) (AUROC for model discriminability, 0.83; 95% CI, .80–.85). (Supplementary Table 3). Subgroup analyses restricted to patients discharged alive from the index hospital stay (aOR, 0.83; 95% CI,.50–1.36) and to migrants (aOR, 1.09; 95% CI, .49–2.41) provided similar results (Supplementary Table 3). VIF values were <1.5 for all variables.

## DISCUSSION

In this French multicenter study, 1 of 7 PHIV admitted to the ICU was facing social precariousness, with undocumented migrants accounting for the largest subgroup. Precarious and nonprecarious PHIV differed markedly regarding the characteristics of HIV and types of critical illnesses. Still, precariousness was not independently linked with an increased hazard of death during the index hospital stay or at 1 year after adjustment on potential confounders.

The number of undocumented migrants admitted to the ICU in western countries has been rising steadily over the past decade, alongside the growing population displacements driven by economic, political, or ecological insecurity [25]. In this subgroup, the prevalence of HIV is up to 6-fold higher than in the general ICU population, likely because of recurrent exposure to the risk of HIV acquisition and limited access to preventive medicine before, during, and after migration [17, 19]. In this cohort, migrants were younger and had fewer AIDS-unrelated comorbidities than other PHIV, a finding that may be related to the physical condition and health status required to undertake and complete the journey from their country of origin (“healthy immigrant paradox”). Importantly, the prevalence of inaugural admissions was substantially higher—and the proportion of patients previously receiving cART lower—in undocumented migrants than in other PHIV, reflecting socioeconomic barriers for inclusion in screening programs, earlier diagnosis of seropositivity, and sustained HIV care. These features directly translated into a disproportioned frequency of admissions for AIDS-related opportunistic infections, whereas such presentations have become quite rare in western ICUs in the late cART era [2, 4, 26]. Importantly, migrant women with HIV appear as a priority target for dedicated healthcare programs as they represented more than half of patients in this subgroup, contrasting with the sharp male predominance usually observed in cohorts of critically ill PHIV [2, 4, 26, 27]. Social issues precluding hospital discharge and protracted workup following the discovery of HIV infection may have contributed to extended lengths of stay in undocumented migrants compared to other PHIV.

Precarious patients other than migrants made up an heterogenous subgroup of PHIV facing poverty, homelessness, or other aspects of socioeconomic deprivation. Compared to nonprecarious PHIV, they presented more frequently with a history of AIDS-defining conditions and, despite similar cART exposure, a lower baseline CD4 cell count and a lesser rate of viral suppression. These features indicate that HIV infection was diagnosed at a more advanced stage, with subsequent hindrance to long-term cART adherence, as previously observed in noncritically ill precarious PHIV [8, 9, 12]. Next, admissions for bacterial sepsis were more common in these patients, which may have several potential explanations. First, poor immunological control is associated with an increased hazard of bacterial infections in PHIV [28]. Second, precariousness in itself constitutes a risk factor for bacterial infections owing to insufficient preventive health care and vaccine coverage, malnutrition, and unsanitary housing [29, 30]. Last, precariousness may delay hospital referring and the initiation of appropriate care (eg, empirical antimicrobial agents, source control when required), thereby easing progression toward sepsis or septic shock [31].

The short-term outcome of critically ill PHIV has been extensively reappraised over the recent years and depends chiefly on age, chronic conditions (especially liver diseases and neoplasms), the extent of organ failures at ICU admission and, though debated, the depth of immune deficiency [3–5, 27]. Yet, whether precariousness exerts an independent impact on hospital survival in this population has not been investigated so far. Long-lasting impediments in access to both primary and specialized care may intuitively expose precarious PHIV with critical illness to worst outcomes than their nonprecarious counterparts. However, in our work, precariousness was not independently linked with an increased hazard of in-hospital death, including in analyses restricted to undocumented migrants. Interestingly, in studies not focused on PHIV, the outcome of critical illness has been shown to correlate with macro-indicators of neighborhood socioeconomic deprivation and local healthcare availability [20, 32, 33], but less consistently with individual markers of poverty [20, 21], notably in migrants [19] and homeless people [34, 35]. Of note, no difference was observed between groups regarding the implementation of organ support or treatment limitation decisions. These data indicate that, for a given degree of clinical severity, precarious and nonprecarious PHIV admitted to the ICU share the same short-term prognosis and predictors of in-hospital death. Importantly, the in-hospital mortality rate that we observed in our study was similar to those currently reported in critically ill PHIV in France and other high-income countries [2, 4, 26, 27, 36], strengthening the external validity of our results. Universal health insurance coverage in France, progress in life-sustaining therapies, policies of early ICU admission, and gradual improvement in intensivists’ knowledge on HIV care may all have mitigated the impact of precariousness on patient outcomes [1, 21].

Overall 1-year mortality in this cohort was consistent with the rates reported in other populations of PHIV discharged alive from the hospital, regardless of whether or not they were admitted to the ICU during the index stay [37]. The available evidence suggests that postdischarge mortality in PHIV increases in those with low CD4 cell count, lack of subsequent specialized follow-up, and unsustained cART adherence [38–40]. Though these features are more common in precarious PHIV, no association was observed between precariousness and the likelihood of death at 1 year in PHIV discharged alive from the ICU. Certain characteristics of the precarious PHIV included in this study—namely, good prior functional status, intermediate severity indexes at admission, low requirement for invasive organ support, relatively short lengths of ICU stay and, for undocumented migrants, a limited burden of comorbidities—may explain these findings as they have been associated with improved long-term survival in the general ICU population [41–43].

This work has limitations that mainly ensue from its retrospective design. First, despite careful adjustment on clinically relevant cofactors, we cannot rule out residual confounding in analyses addressing the study endpoints. Next, patients were classified as precarious or nonprecarious according to the information available in the medical charts. This approach might have led to misclassification and underestimation of the actual number of precarious PHIV, which could have blurred a difference between the 2 groups regarding short- and long-term mortality. The prospective use of granular social deprivation indexes could have allowed a more precise assessment of the relationship between precariousness and outcomes [21, 44]. Along this line, we did not collect data on geographic origin, educational level, time elapsed since arrival in France, and actual access to healthcare for undocumented migrants; these features might have affected both the clinical presentation and prognosis of this subgroup. Then, our study might have lacked statistical power to detect a significant association between precariousness and 1-year mortality, as suggested by wide CIs for aOR. Also, long-term cART adherence, viral suppression, and immune recovery were not appraised when analyzing the likelihood of survival at 1 year in ICU survivors. Moreover, precarious patients lost to follow-up at 1 year could have been those facing the most severe barriers for access to health care. Last, we did not investigate the effect of precariousness on the progression of AIDS-unrelated chronic diseases or the occurrence of postintensive care syndrome in PHIV surviving critical illness.

## CONCLUSION

Precarious and nonprecarious PHIV requiring ICU admission have distinct clinical features that likely reflect chronic inequities in access to HIV care, emphasizing the need for tailored health interventions. However, precariousness was not independently linked with a higher hazard of death during the index hospital stay or at 1 year, though our study might have been underpowered to investigate this latter outcome.

## Supplementary Material

ofaf687_Supplementary_Data

## Supplementary Data

Supplementary materials are available at *Open Forum Infectious Diseases* online. Consisting of data provided by the authors to benefit the reader, the posted materials are not copyedited and are the sole responsibility of the authors, so questions or comments should be addressed to the corresponding author.
