# Supplementary material for: Social Precariousness and the Outcome of Critical Illnesses in People with HIV: A Multicenter Cohort Study
Source: Open Forum Infect Dis. 2025 Nov 12;12(12):ofaf687. doi: 10.1093/ofid/ofaf687 (PMC12661657; doi:10.1093/ofid/ofaf687)
Supplement: ofaf687_Supplementary_Data [file ofaf687_supplementary_data.docx]

**ELECTRONIC SUPPLEMENTARY MATERIAL**

**Social precariousness and the outcome of critical illnesses in people living with HIV: a multicenter cohort study**

Piotr Szychowiak, MD; Thierry Boulain, MD; Étienne de Montmollin, MD, PhD; Jean-François Timsit, MD, PhD; Alexandre Elabbadi, MD; Laurent Argaud, MD, PhD; Stephan Ehrmann, MD, PhD; Nahema Issa, MD; Emmanuel Canet, MD, PhD; Frédéric Martino, MD; Fabrice Bruneel, MD; Jean-Pierre Quenot, MD, PhD; Florent Wallet, MD; Élie Azoulay, MD, PhD; François Barbier, MD, PhD

**Precariousness: definitions and classification procedure**

All the data exposed and analyzed in this research were extracted from anonymized medical charts provided by local investigators for the first study conducted on this cohort (1), except those regarding precariousness.

For the present work, two of the study coordinators (P.S. and F.B.) identified indicators of precariousness through a second round of reviewing of the medical charts. In the participating ICUs as in most ICUs in France, medical charts include a section dedicated to the patient’s family status, country of origin, and socioeconomic level with information regarding housing, profession, and potential social assistance. These features are routinely collected at ICU admission since they may help understanding the patients’ medical history and improve communication with their relatives during the ICU stay. Precariousness was defined according to three of the five domains of social determinants of health proposed by the Office of Disease Prevention and Health Promotion (US Department of Health and Human Services) (2), namely economic stability, neighborhood and built environment, and social and community context. The fourth domain related to education access and quality was not reliably assessable through reviewing of medical records. The fifth domain related to health care access and quality was the purpose of the study.

Undocumented migrants were defined as international immigrants without permanent residence permit for living in France such as refugees and asylum seekers (3).

Precarious patients other than undocumented migrants were defined as patients falling into at least one the following, non-mutually exclusive categories:

- Homelessness: lack of stable housing with nights routinely spent in temporary shelters or in places not intended for habitation (*e.g*., street, station, or squat)
- Incarceration (*i.e*., in jail at the time of ICU admission)
- Poor housing: living in insalubrious, unheated and/or overcrowded habitation due to economic instability
- Social isolation: lack of social support combined with economic instability
- Social marginalization: systematic exclusion based on societal or economic norms and resulting in social disadvantages, including inequity in access to health policies (4)

References

1. Szychowiak P, Boulain T, Timsit JF, Elabbadi A, Argaud L, Ehrmann S, et al. Clinical spectrum and prognostic impact of cancer in critically ill patients with HIV: a multicentre cohort study. Ann Intensive Care. 2023 Aug 22;13(1):74.

2. US Department of Health and Human Services, Office of Disease Prevention and Health Promotion. Social determinants of health [Internet]. Available from: www.odphp.health.gov/healthypeople/priority-areas/social-determinants-health

3. Hraiech S, Papazian L, Azoulay E. Migrants in the intensive care unit: time to show we care. Intensive Care Med. 2021 Apr;47(4):473–5.

4. Fluit S, Cortés-García L, von Soest T. Social marginalization: A scoping review of 50 years of research. Humanit Soc Sci Commun. 2024 Dec 18;11(1):1665.

**Patient characteristics entered in the multivariable logistic models**

The following patient characteristics were entered in the multivariable logistic models built to identify independent predictors or in-hospital and one-year mortality, before augmented backward elimination (see the statistical paragraph of the article for methodological aspects):

- Age
- Sexe
- Alcohol addiction
- Illicit drug addiction
- WHO performance status
- Diabetes mellitus
- Chronic obstructive pulmonary disease
- Chronic respiratory disease other than chronic obstructive pulmonary disease
- Chronic cardiac disease
- Liver cirrhosis
- Chronic kidney disease without chronic hemodialysis
- Chronic hemodialysis
- Chronic neurological disease
- Chronic psychiatric disease
- Solid organ transplantation
- Solid non-AIDS-defining cancer
- Hematological non-AIDS-defining cancer
- Hematological AIDS-defining cancer
- Kaposi sarcoma
- Newly diagnosed HIV infection
- History of AIDS-defining opportunistic infections
- Use of combination antiretroviral therapy before ICU admission
- SAPS2 value at ICU admission
- SOFA score value at ICU admission
- Bacterial sepsis as main diagnosis of the ICU stay
- AIDS-defining opportunistic infections as main diagnosis of the ICU stay
- Exacerbation of non-AIDS-related/non-cancer-related chronic condition as main diagnosis of the ICU stay
- Other non-AIDS-related diagnosis as main diagnosis of the ICU stay
- ARDS during the ICU stay
- Invasive mechanical ventilation during the ICU stay
- Vasopressors during the ICU stay
- Renal replacement therapy during the ICU stay
- Precariousness (all – undocumented migrants and other precarious patients)
- Undocumented migrants

**Table S1.** Types of social deprivation in precarious patients

| **Type of precariousness** | **Patients**  (n = 136) |
| --- | --- |
| **Undocumented migrants** | **54/136 (39.7)** |
| **Precarious patients other than undocumented migrants**  Homeless  Incarcerated  Other forms of social deprivation ^a^  Poor housing  Social isolation  Social marginalization  Sex worker | **82/136 (60.3)**  29/82 (35.4)  1/82 (1.2)  52/82 (63.4)  40/82 (48.8)  26/82 (31.7)  10/82 (12.2)  3/82 (3.7) |

*Table S1 footnote*

Data are exposed as number (percentage).

^a^ Individuals could fall into more than one category.

**Table S2.** Characteristics of the study population according to the vital status at hospital discharge

|  | **All patients**  (n = 939) | **Alive at hospital discharge**  (n = 772) | **Deceased at hospital discharge**  (n = 167) | ***P*-value** |
| --- | --- | --- | --- | --- |
| **Male sex** | 670 (71.3) | 551 (71.4) | 119 (71.3) | 1 |
| **Age, years** | 52 (43-59) | 51 (42-59) | 55 (48-63) | <0.0001 |
| **Precariousness** ^a^ | 136 (14.5) | 111 (14.4) | 25 (15.0) | 0.81 |
| **Addiction (current or past)**  Tobacco  Alcohol  Drug  IVDU | 305 (32.5)  195 (20.8)  180 (19.2)  72 (7.7) | 266 (34.5)  167 (21.6)  157 (20.3)  62 (8.0) | 39 (23.4)  28 (16.8)  23 (13.8)  10 (6.0) | 0.007  0.19  0.06  0.43 |
| **Chronic conditions**  Diabetes mellitus  Respiratory  COPD  Cardiac  Hepatic  HBV co-infection  HCV co-infection  Liver cirrhosis  Renal  Chronic hemodialysis  Neurological  Solid organ transplantation  Active AIDS-defining cancer  Hematological/NHL  Solid/Kaposi sarcoma  Solid/Cervix cancer  Active non-AIDS-defining cancer  Hematological  Solid | 134 (14.3)  199 (21.2)  107 (11.4)  175 (18.6)  200 (21.3)  72 (7.7)  111 (11.8)  74 (7.9)  171 (18.2)  30 (3.2)  105 (11.2)  24 (2.6)  106 (11.7)  86 (9.2)  19 (2.0)  1 (0.1)  97 (7.1)  35 (3.7)  62 (6.6) | 105 (13.6)  171 (22.2)  89 (11.5)  143 (18.5)  161 (20.9)  58 (7.5)  89 (11.5)  50 (6.5)  136 (17.6)  24 (3.1)  90 (11.7)  20 (2.6)  74 (9.6)  59 (7.6)  14 (1.8)  1 (0.1)  53 (6.9)  24 (3.1)  29 (3.7) | 29 (17.4)  28 (16.8)  18 (10.8)  32 (19.2)  39 (23.4)  14 (8.4)  22 (13.2)  24 (14.4)  35 (21.0)  6 (3.6)  15 (9.0)  4 (2.4)  32 (19.2)  27 (16.2)  5 (3.0)  0  44 (26.3)  11 (5.6)  33 (19.8) | 0.25  0.14  0.89  0.83  0.47  0.82  0.64  0.001  0.37  0.94  0.42  1  0.001  0.001  0.36  1  <0.0001  0.04  <0.0001 |
| **WHO performance status**  0-2  3-4 | 848 (91.3)  91 (9.7) | 711 (92.1)  61 (7.9) | 137 (82.0)  30 (18.0) | 0.0002 |
| **HIV-related characteristics**  Newly diagnosed HIV infection ^b^  CD4 cell count at admission, per µL ^c^  HIV viral load at admission, per µL ^d^  Previously known HIV infection  cART at hospital admission  Baseline CD4 cell count, per µL ^e^  Baseline HIV viral load, per µL ^f^  History of AIDS-defining OI  History of HIV encephalitis  History of Castleman disease  History of ADC (remission or cured)  NHL  Kaposi sarcoma  Cervix cancer | 127 (13.5)  51 (20-147)  5.10^5^ (10^5^-10^6^)  812 (86.5)  699 (74.4)  370 (180-600)  <50 (<50-<50)  301 (32.1)  21 (2.2)  16 (1.7)  50 (5.3)  11 (1.2)  39 (4.2)  1 (0.1) | 105 (13.6)  55 (20-158)  5.10^5^ (10^5^-10^6^)  667 (86.4)  572 (74.1)  392 (198-603)  <50 (<50-75)  240 (31.1)  17 (2.2)  13 (1.7)  40 (5.2)  7 (0.9)  32 (4.1)  1 (0.1) | 22 (13.2)  34 (22-123)  10^6^ (10^5^-10^6^)  145 (86.8)  127 (76.0)  280 (122-500)  <50 (<50-50)  61 (36.5)  4 (2.4)  3 (1.8)  10 (6.0)  4 (2.4)  7 (4.2)  0 | 1  0.57  0.55  1  0.63  0.005  0.25  0.17  1  1  0.67  0.11  0.98  1 |
| **McCabe score**  0  1  2 | 695 (74.0)  189 (20.1)  55 (5.9) | 603 (78.1)  137 (17.7)  32 (4.1) | 92 (55.1)  52 (31.1)  23 (13.8) | <0.0001 |

**Table S2 (continued).**

|  | **All patients**  (n = 939) | **Alive at hospital discharge**  (n = 772) | **Deceased at hospital discharge**  (n = 167) | ***P*-value** |
| --- | --- | --- | --- | --- |
| **Type of ICU admission**  Medical  Unscheduled surgery  Scheduled surgery | 904 (96.3)  13 (1.4)  22 (2.3) | 743 (96.2)  11 (1.4)  18 (2.3) | 161 (96.4)  2 (1.2)  4 (2.4) | 0.97 |
| **Direct ICU admission**  Direct ^g^  Transfer from wards | 603 (64.2)  336 (35.8) | 529 (68.5)  243 (31.5) | 74 (44.3)  93 (55.7) | <0.0001 |
| **SAPS 2 at ICU admission** | 36 (26-51) | 34 (24-46) | 51 (39-69) | <0.0001 |
| **SOFA score at ICU admission** | 4 (2-6) | 3 (1-5) | 7 (4-12) | <0.0001 |
| **Main reason for ICU admission**  Acute respiratory failure  Sepsis/septic shock  Coma (non-toxic)  Acute kidney failure  Metabolic  Drug overdose  Shock (other than septic)  Cardiac arrest  Others | 320 (34.1)  172 (18.3)  165 (17.6)  56 (6.0)  47 (5.0)  47 (5.0)  28 (3.0)  16 (1.7)  88 (9.3) | 265 (34.3)  129 (16.7)  142 (18.4)  48 (6.2)  38 (4.9)  47 (6.1)  18 (2.3)  4 (0.5)  81 (10.6) | 55 (32.9)  43 (25.7)  23 (13.8)  8 (4.8)  9 (5.4)  0  10 (6.0)  12 (7.2)  7 (4.2) | <0.0001 |
| **Neutropenia at ICU admission** | 46 (4.9) | 31 (4.0) | 15 (9.0) | 0.02 |
| **Organ support in the ICU**  High-flow nasal oxygen therapy  Non-invasive ventilation  Invasive mechanical ventilation  Vasopressors  Renal replacement therapy  VA-ECMO  VV-ECMO | 96 (10.2)  73 (7.8)  301 (32.1)  242 (25.8)  105 (11.2)  4 (0.4)  10 (1.1) | 72 (9.3)  59 (7.6)  191 (24.7)  144 (18.7)  61 (7.9)  2 (0.3)  3 (0.4) | 24 (14.4)  14 (8.4)  110 (65.9)  98 (58.7)  44 (26.3)  2 (1.2)  7 (4.2) | 0.07  0.75  <0.0001  <0.0001  <0.0001  0.15  0.0004 |
| **Main diagnosis of the ICU stay**  Bacterial sepsis  AIDS-defining opportunistic infection  *Pneumocystis jirovecii* pneumonia  Cerebral toxoplasmosis  Tuberculosis  Other OI  AIDS-defining cancer  Non-AIDS-defining cancer  Exacerbation of comorbidities other than cancer  Miscellaneous | 263 (28.0)  156 (16.6)  69 (7.3)  30 (3.2)  14 (1.5)  43 (4.6)  69 (7.3)  47 (5.0)  242 (25.8)  162 (17.3) | 202 (26.2)  136 (17.6)  60 (7.8)  26 (3.4)  12 (1.6)  38 (4.9)  47 (6.1)  27 (3.5)  222 (28.8)  138 (17.9) | 61 (36.5)  20 (12.0)  9 (5.4)  4 (2.4)  2 (1.2)  5 (3.0)  22 (13.2)  20 (12.0)  20 (12.0)  24 (14.4) | 0.008  0.08  0.63  0.003  <0.0001  <0.0001  0.31 |
| **Chemotherapy in the ICU** | 71 (7.6) | 46 (6.0) | 25 (15.0) | 0.0003 |
| **Adverse events during the ICU stay**  Ventilator-associated pneumonia  VAPA  Infection or colonization with MDRB  *Clostridioides difficile* infection  ARDS  Cardiac arrest | 54 (5.8)  4 (0.4)  121 (12.9)  13 (1.4)  124 (13.2)  31 (3.3) | 27 (3.5)  1 (0.1)  92 (11.9)  10 (1.3)  58 (7.5)  7 (0.9) | 27 (16.2)  3 (1.8)  29 (17.4)  3 (1.8)  66 (39.5)  24 (14.4) | <0.0001  0.02  0.001  0.89  <0.0001  <0.0001 |

**Table S2 (continued).**

|  | **All patients**  (n = 939) | **Alive at hospital discharge**  (n = 772) | **Deceased at hospital discharge**  (n = 167) | ***P*-value** |
| --- | --- | --- | --- | --- |
| **TLD during the ICU stay**  Organ support withdrawal  Organ support withholding | 110 (11.7)  38 (4.0)  72 (7.7) | 27 (3.5)  0  27 (3.5) | 83 (49.7)  38 (22.8)  45 (26.9) | <0.0001  <0.0001  <0.0001 |
| **ICU readmission** | 48 (5.3) | 37 (5.0) | 11 (6.6) | 0.53 |
| **Outcomes**  ICU length of stay, days  Hospital length of stay, days  In-ICU death | 5 (3-9)  19 (10-36)  112 (11.9) | 5 (3-8)  18 (10-35)  - | 7 (3-16)  20 (10-40)  112 (67.1) | <0.0001  0.52  NA |

*Table S2 footnote*

Data are exposed as number (percentage) or median (interquartile range).

WHO, World Health Organization; cART, combination antiretroviral therapy; OI, opportunistic infection; ADC, AIDS-defining cancer; NHL, non-Hodgkin lymphoma; IVDU, intravenous drug use; COPD, chronic obstructive pulmonary disease; CAD, coronary heart disease; NADC, non-AIDS-defining cancer; ICU, intensive care unit; SAPS 2, simplified acute physiology score 2; SOFA, sepsis-related organ failure assessment; OI, opportunistic infection; AKI, acute kidney injury; VA/VV-ECMO, veino-arterial/veino-veinous extracorporeal membrane oxygenation; VAPA, ventilator-associated pulmonary aspergillosis; MDRB, multidrug-resistant bacteria; ARDS, acute respiratory distress syndrome

^a^ Homeless (n = 29), undocumented migrants (n = 54), incarcerated patient (n = 1) and other forms of social deprivation (n = 52); ^b^ Diagnosis of HIV infection during the same hospital stay (inaugural admission); ^c^ Missing value for 16/127 patients with newly diagnosed HIV infection; ^d^ Missing value for 22/127 patients with newly diagnosed HIV infection; ^e^ Within 6 months prior to ICU admission, missing values for 273/812 patients with previously known HIV infection; ^f^ Within 6 months prior to ICU admission, missing values for 196/812 patients with previously known HIV infection; ^g^ ICU admission within the first 24 hours following hospital admission

**Table S3.** Independent predictors of one-year mortality: results of final models

|  | **All patients** | |  | **Patients alive at hospital discharge** | |  | **Migrants and non-precarious patients only** | |  |
| --- | --- | --- | --- | --- | --- | --- | --- | --- | --- |
|  | aOR (95% CI) | *P*-value |  | aOR (95% CI) | *P*-value |  | aOR (95% CI) | *P*-value |  |
| Male sex | 1.06 (0.73-1.55) | 0.76 |  | 1.03 (0.71-1.51) | 0.87 |  | 1.00 (0.67-1.49) | 0.98 |  |
| Age, per 10-year increase | 1.13 (0.96-1.33) | 0.13 |  | 1.15 (0.99-1.35) | 0.07 |  | 1.15 (0.97-1.36) | 0.12 |  |
| Alcohol addiction | - | - |  | 0.99 (0.65-1.50) | 0.95 |  | - | - |  |
| Drug addiction | 0.68 (0.42-1.10) | 0.11 |  | - | - |  | 0.72 (0.42-1.22) | 0.22 |  |
| Diabetes mellitus | 2.11 (1.32-3.38) | 0.002 |  | 1.86 (1.17-2.95) | 0.009 |  | 2.06 (1.26-3.36) | 0.004 |  |
| COPD | 2.21 (1.29-3.80) | 0.004 |  | 1.79 (1.07-3.01) | 0.03 |  | 2.43 (1.37-4.32) | 0.002 |  |
| Liver cirrhosis | 2.74 (1.53-4.91) | 0.0007 |  | 2.39 (1.34-4.26) | 0.003 |  | 3.18 (1.74-5.80) | 0.0002 |  |
| CKD without chronic hemodialysis | 1.22 (0.76-1.97) | 0.41 |  | 1.11 (0.69-1.78) | 0.67 |  | 1.32 (0.80-2.16) | 0.27 |  |
| Chronic hemodialysis | 3.82 (1.57-9.30) | 0.003 |  | 3.79 (1.61-8.92) | 0.002 |  | 3.15 (1.20-8.23) | 0.02 |  |
| Solid NADC | 9.68 (4.85-19.31) | <0.0001 |  | 8.45 (4.28-16.68) | <0.0001 |  | 8.83 (4.27-18.28) | <0.0001 |  |
| Hematological NADC | 2.59 (1.16-5.79) | 0.02 |  | - | - |  | 2.62 (1.12-6.16) | 0.03 |  |
| Hematological ADC | 2.50 (1.43-5.35) | 0.0013 |  | 2.72 (1.32-3.85) | 0.004 |  | 2.86 (1.59-5.26) | 0.0005 |  |
| Kaposi sarcoma | 2.42 (0.86-6.83) | 0.09 |  | 4.01 (1.53-10.54) | 0.005 |  | 3.21 (1.04-9.94) | 0.04 |  |
| WHO performance status | 1.59 (1.36-1.86) | <0.0001 |  | 1.53 (1.31-1.78) | <0.0001 |  | 1.62 (1.37-1.92) | <0.0001 |  |
| Newly diagnosed HIV infection | 0.71 (0.41-1.24) | 0.23 |  | 0.81 (0.47-1.39) | 0.45 |  | 0.65 (0.36-1.17) | 0.15 |  |
| SOFA score value at ICU admission | 1.15 (1.09-1.20) | <0.0001 |  | 1.20 (1.14-1.25) | <0.0001 |  | 1.14 (1.09-1.21) | <0.0001 |  |
| RRT during the ICU stay | - | - |  | 1.02 (0.59-1.77) | 0.94 |  | - | - |  |
| Exacerbation of chronic condition as main diagnosis of the ICU stay | 0.65 (0.41-1.03) | 0.07 |  | 0.79 (0.54-1.17) | 0.24 |  | 0.67 (0.41-1.08) | 0.10 |  |
| Bacterial sepsis as main diagnosis of the ICU stay | 0.70 (0.45-1.09) | 0.11 |  | - | - |  | 0.68 (0.43-1.09) | 0.11 |  |
| **Precariousness** | **0.89 (0.54-1.48)** | **0.66** |  | **0.83 (0.50-1.36)** | **0.46** |  | **-** | **-** |  |
| **Precariousness (i.e., migrants)** | **-** | **-** |  | **-** | **-** |  | **1.09 (0.49-2.41)** | **0.83** |  |

*Table S3 footnote*

aOR, adjusted odd ratio; CI, confidence interval; COPD, chronic obstructive pulmonary disease; CKD, chronic kidney disease; ADC/NADC, AIDS-defining/non-AIDS-defining cancer; WHO, World Health Organization; SAPS-2, simplified acute physiology score; ICU, intensive care unit; SOFA, sepsis-related organ failure assessment; ARDS, acute respiratory distress syndrome; RRT, renal replacement therapy

**Figure S1.** Study flowchart

952 patients with HIV with a first admission in the 12 participating ICUs over the inclusion period

(January 2015 – June 2020)

Exclusion due to missing essential data:

cART use at ICU admission, n = 3

Vital status at hospital discharge, n = 10

**939 patients included in**

**the study cohort**

**136 precarious patients (14.5%)**

including 54 migrants (5.8%) and 82 patients other than migrants (8.7%)

**803 non-precarious patients (85.5%)**

*Figure S1 footnote*

ICU, intensive care unit; cART, combination antiretroviral therapy

**Figure S2.** Proportions of precarious and non-precarious critically ill patients with HIV in the participating intensive care units

*Figure S2 footnote*

ICU, intensive care unit

Numbers within brackets indicate the total number of patients included in each ICU.

*P* = 0.80 (inter-ICU comparison, χ^2^ test)

**Figure S3.** In-hospital mortality rates in precarious and non-precarious critically ill patients with HIV according to the main diagnosis of the ICU stay

*Figure S3 footnote*

Data are exposed as percentages.

*P* >0.05 for all comparisons between precarious and non-precarious patients.

Note that admissions for miscellaneous reasons are not exposed due to the low number of patients.
